# Stretchable MXene/Thermoplastic Polyurethanes Based Strain Sensor Fabricated Using a Combined Electrospinning and Electrostatic Spray Deposition Technique

**DOI:** 10.3390/mi12030252

**Published:** 2021-03-01

**Authors:** Feiyu Fang, Han Wang, Huaquan Wang, Xiaofei Gu, Jun Zeng, Zixu Wang, Xindu Chen, Xin Chen, Meiyun Chen

**Affiliations:** 1 State Key Laboratory of Precision Electronic Manufacturing Technology and Equipment, School of Electromechanical Engineering, Guangdong University of Technology, Guangzhou 510006, China; feiyu93@foxmail.com (F.F.); fungomail@126.com (J.Z.); wangzixu0511@gmail.com (Z.W.); chenxindu@gdut.edu.cn (X.C.); chenx@gdut.edu.cn (X.C.); 2 Ji Hua Laboratory (Advanced Manufacturing Science and Technology GuangDong Laboratory), Foshan 528200, China; 3 Guangdong Provincial Key Laboratory of Micro-Nano Manufacturing Technology and Equipment, School of Electromechanical Engineering, Guangdong University of Technology, Guangzhou 510006, China; 4 China Resources Cement Technology Research and Development CO. LTD, Guangzhou 510460, China; whq931@163.com; 5 Department of Stomatology, The Third Affiliated Hospital of Sun Yat-sen University, Guangzhou 510630, China; xiaofeisysu@foxmail.com

**Keywords:** strain sensor, electrospinning, electrostatic spray deposition, MXene

## Abstract

In this work, a novel flexible electrically resistive-type MXene/Thermoplastic polyurethanes(TPU) based strain sensors was developed by a composite process of electrospinning (ES) and electrostatic spray deposition (ESD). Compared with other deposition processes, the sensing layer prepared by ESD has better adhesion to the ES TPU nanofiber membrane and is not easy to crack during the stretching process, thereby greatly improving the working range of the strain sensor. Furthermore, we obtained the sandwich structure easily by ES on the surface of the sensing layer again. This will help make the stress distribution more uniform during the stretching process and further increase the strain sensing range. The ESD-ES strain sensors were attached on skin to monitor various human motions. The results demonstrate that our ESD-ES strain sensors have wide application prospects in smart wearable device.

## 1. Introduction

Stretchable and wearable strain sensors have gained great interest due to their portability, foldability and portability. It is widely used for health monitoring and motion detection [1,2,3,4,5]. However, most of the reported strain sensors are not capable of achieving both high sensitivity and broad sensing range, limiting their application [6,7,8,9,10]. Strain sensors based on one-dimensional conductive materials can obtain a larger stretch range, but the sensitivity is reduced. Strain sensors based on two-dimensional conductive materials can achieve higher sensitivity, but the stretching range is limited [7,11]. So far, it is still remains challenging to fabricate strain sensors that have both high sensitivity and broad sensing range [12].

MXenes, a new family of two-dimensional (2D) transition metal carbides and carbonitrides, has metal conductivity, excellent mechanical properties and hydrophilic surface, thus showing great potential in the field of electrochemical energy and development of the next generation high sensitivity strain sensor [7,10,11,12,13]. MXenes reported so far are synthesized by wet-chemical etching in hydrofluoric acid (HF) or HF-forming etchants or HF-containing, which add surface functionalities such as −O, −F, or −OH, represented by T_x_ in this formula as M_n+1_X_n_T_x_ [14]. However, the interaction between MXene sheets is very strong due to the abundant surface functional groups, so the MXene sheets will not easily slide from each other. Therefore, MXenes based strain sensor also faces the problem of a narrow stretch range caused by the rapid crack propagation of stacked MXene sheets rather than the slippage one, which significantly limits the application of MXene-based sensors [7,12,15].

Electrospinning (ES) is an efficient nanofiber production method with many advantages such as simple process and low cost [16]. The nanofibers prepared by ES have the characteristics of large specific surface area and high porosity. With the above advantages, electrospinning has been widely used in many fields such as biomedicine, flexible electronics, and filtration membranes [8,17,18,19].

Electrostatic spray deposition (ESD) is a simple, low-cost, efficient, and flexible atomization deposition process, by which the atomized droplets can be easily controlled at the micro-nano scale [20,21,22,23,24]. Compared with other thin film deposition technologies, such as chemical vapor deposition (CVD) or physical vapor deposition (PVD), the deposition rate of ESD technology is higher. Moreover, electrospray is a simple and non-vacuum-based technique and it can work at room temperature, allowing a wider range of substrate materials that can be coated [20].

In this study, Ti_3_C_2_T_x_ MXene based strain sensors with high sensitivity and broad sensing range was fabricated through the ESD-ES composite process, which also showed satisfactory repeatability in 1000 times stretch-release cycles test. The electrospun TPU porous nanofiber membrane is used as a flexible, stretchable and breathable substrate to introduce excellent stretchability and breathability, while the Ti_3_C_2_T_x_ MXene coating layer deposition by ESD process is used as the strain sensing layer. Most importantly, benefiting from the unique advantages of the ESD process, the workable strain range has been greatly improved (from 19% to 42%) without the addition of other one-dimensional conductive materials, compared to the Ti_3_C_2_T_x_ MXene sensor prepared by the drop-coated method. On the other hand, we encapsulated the sensing layer by electrospinning the TPU nanofiber several times to prepare a strain sensor with a sandwich structure. This is conducive to uniform strain distribution, thereby further increasing the workable strain to more than 72%.

## 2. Experimental Section

### 2.1. Preparation of Ti3C2Tx MXene and Ti3C2Tx Inks

**Figure 1:**
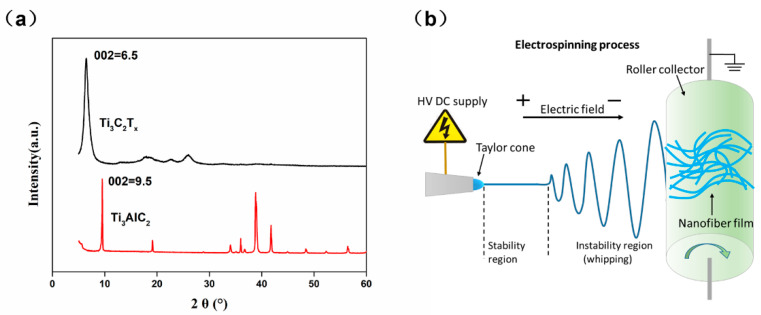
(**a**) XRD patterns of Ti_3_C_2_Tx and Ti_3_AlC_2_; (**b**) Schematic diagram of electrospinning.

Ti_3_C_2_Tx MXene was synthesized by the most frequently used method of chemical liquid etching as previously published works [13,14]. Specifically, 2 g Ti_3_AlC_2_ was added to a mixture of 40 mL HCl solution (37 wt%, Sinopharm, China) and 2 g LiF (Sigma-Aldrich, USA), and the mixture was continuously stirred in a PTFE container at 35 °C for 24 h. The obtained suspension was washed several times with deionized water, and centrifuged with a centrifuge (centrifugal speed 3500 rpm/min) until the pH value was 6, to obtain multilayer Ti_3_C_2_T_x_ (m-Ti_3_C_2_T_x_) precipitate. Then under ice bath conditions, the multilayer Ti_3_C_2_T_x_ MXene (m-Ti_3_C_2_T_x_) was ultrasonically dispersed in deionized water. After centrifugation, stable multilayer Ti_3_C_2_T_x_ Mxene suspension (delaminated Ti_3_C_2_T_x_, d-Ti_3_C_2_T_x_) can be obtained. The suspension was suction filtered, and then freeze-dried to obtain a small layer of Ti_3_C_2_T_x_ MXene product (d-MXene). Finally, an appropriate amount of d-MXene was ultrasonically dispersed in IPA (Isopropyl alcohol, Aladdin, China) to prepare Ti_3_C_2_T_x_ conductive ink with a concentration of 10 mg/mL. Figure 1a shows the X-ray diffraction (XRD) patterns of the Ti_3_C_2_T_x_.

### 2.2. Fabrication of TPU Nanofiber Membranes

TPU Nanofiber Membranes were fabricated using electrospinning. Thermoplastic polyurethane (TPU) was dissolve in a 1:1 N,N-dimethylformamide (DMF): acetone (*v*/*v*) solution (total concentration of solution, 20 wt%). TPU was supplied by Hongye Co., Ltd. (Shenzhen, China). N,N-dimethylformamide (DMF), and acetone were purchased from Sinopharm Chemical Reagent Co., Ltd.(Shanghai, China). The TPU solution for electrospinning was stirred at 30 °C for 24 h. The electrospinning equipment was purchased from Qingzi Nano Corp. (Foshan, China). Figure 1b shows schematic diagram of electrospinning.

The syringe sucks 3 mL spinning solution and installs it on the precision syringe pump. The nozzle used for electrospinning is a 22G dispensing nozzle (inner diameter 420 μm and external diameter 720 μm), and it is connected to the positive pole of the high-voltage power supply. Stick a conductive aluminum foil tape of appropriate size on the roller as a receiving substrate and ground the roller well. Adjust the distance between the spinning nozzle and the roller to 10 cm, and set the roller speed to 1000 rpm/min. Set the flow rate of the precision syringe pump to 1.5 ml/h, turn on the high-voltage power supply, set the voltage to 12 KV, and start spinning until the 3 mL TPU solution is completely consumed.

### 2.3. Electrostatic Spray Deposition MXene Sensing Layer

**Figure 2:**
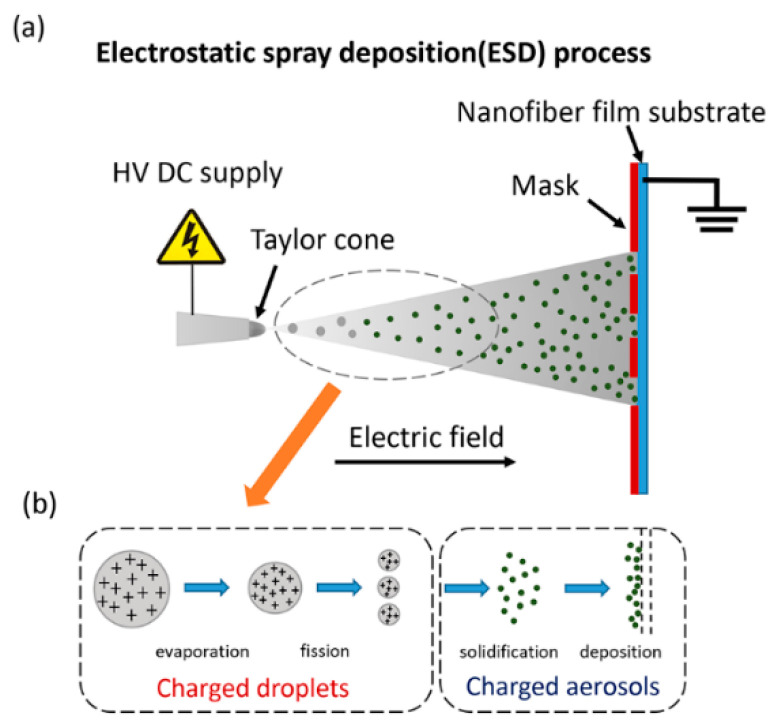
(**a**) Schematic diagram of electrostatic spray deposition; (**b**) Schematic diagram of charged aerosols generation and deposition process.

The device of electrostatic spray deposition is the same as that of electrospinning. Schematic diagram of electrostatic spray deposition as shown in Figure 2. Use a syringe to draw 2 mL Ti_3_C_2_T_x_ Inks and install it on a precision syringe pump. The nozzle used for ESD is a 22G needle and connect it to the positive pole of the high-voltage power supply. After the TPU film is obtained by electrospinning, there is no need to peel it off the roller. The hollow patterned polyethylene terephthalate (PET) template is tightly covered on the surface of the TPU electrospun fiber membrane on the roller. The distance between the nozzle and the roller is 6 cm, and the roller speed is 60 rpm/min. The flow rate of the precision syringe pump is 1 mL/h. The voltage of high-voltage power supply is 14 kV. Until 2 mL Ti_3_C_2_T_x_ inks is completely consumed, stop the ESD process. It can be observed that there is a black substance deposit in the hollow of the template on the surface of the TPU fiber membrane after ESD.

### 2.4. Design and Fabrication of the Strain Sensor

**Figure 3:**
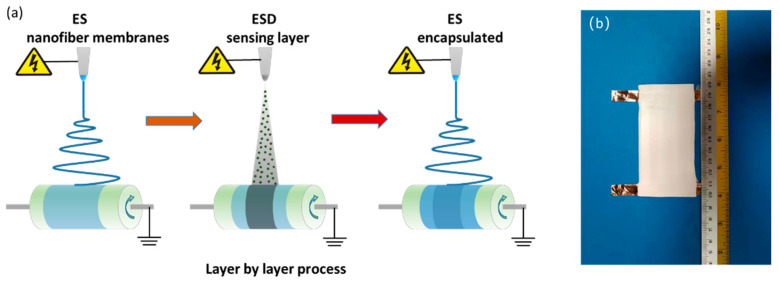
(**a**) Schematic illustration of the fabrication process of the strain sensor; (**b**) Photographs of an ESD-ES strain sensor.

Figure 3a illustrates schematically the preparation procedure of the strain sensor. (1) First prepare and obtain an ES nanofiber membrane; (2) Coated the MXene sensing layer on the surface of ES nanofiber membrane by ESD process. Then use conductive silver paste and copper foil on the surface of the conductive layer to lead out the wire ends. (3) ES is performed again to encapsulate the nanofiber membrane with ESD sensing layer. The flexible nanofiber membrane that with the ESD sensing layer of MXene used as the collection substrate for ES. The newly formed ES nanofibers membrane cover the surface of the conductive layer and are integrated with the original ES flexible nanofibers membrane to realize the sandwich structure assembly, as shown in Figure 3b.

These fabrication processes with room-temperature processing have various advantages including simple operation, little waste, and low cost. On the other hand, we use electrospinning technic to spin the TPU nanofiber on the surface of the sensing layer again, thereby sandwiching the Ti_3_C_2_T_x_ layer between the TPU nanofiber membranes.

For comparison, the Ti_3_C_2_T_x_ MXene sensing layer was also prepared by drop-coated. The process of preparing the sensing layer by the drop-coated method: (1) First prepare and obtain a flexible substrate of electrospun nanofiber; (2) Place the flexible substrate prepared by electrospinning on a glass plate. Use a syringe to suck 2 ml of MXene conductive ink and apply it to the surface of the electrospun film substrate. Let it stand for 3 h and dry naturally to form a thin continuous layer of MXene sheet; then use conductive silver paste and copper foil on the surface of the conductive layer to lead out the wire ends; (3) ES is performed again to encapsulate the nanofiber membrane with drop-coated sensing layer. The flexible nanofiber membrane that with the drop-coated sensing layer of MXene is pasted on the drum and used as the collection substrate for ES.

### 2.5. Characterization

The morphologies of samples were characterized by an Optical microscope (Nikon MM-400, Japan) and SEM (TM3030, Hitachi, Japan). A tensile test machine (Shimadzu AG-X plus, Japan) was used to test the strain sensing characteristics and the electromechanical properties were measured by a digital multimeter (Agilent 34410, Gainesville, GA, USA).

## 3. Results and Discussion

### 3.1. Electrostatic Spray Deposition Process

The typical device of the ESD process (as shown in Figure 2) mainly includes three parts: (1) a metal nozzle, which is connected to the syringe and provides solution; (2) a template and a collection substrate; (3) a high-voltage DC power supply.

The typical ESD process mainly includes the following steps: (1) spray formation; (2) transportation, evaporation, and disruption; (3) the charged aerosol absorbed by the substrate under the action of Coulomb force; (4) The diffusion and penetration of micro-droplets on the surface of the substrate [21]. If the ambient temperature is high enough or the solvent volatilization speed is fast enough, the fine droplets will be fully dried before reaching the substrate to form non-agglomerated discrete nanoparticles, which are adsorbed onto the substrate by the Coulomb force.

During the ESD process, the solution is atomized into an aerosol due to the high-voltage direct current electric field between the nozzle and the substrate. After that, the charged aerosol (consisting of charged droplets) is attracted and deposited on the substrate, which is accompanied by the evaporation of the solvent. In the end, thin film materials with various characteristic shapes are obtained on the substrate. Moreover, in addition to the precursor solution forming a thin film, a particle suspension of the material can also be deposited on the substrate to form a solid layer. A diagram of the steps involved in micro- and nano-particle production is shown in Figure 2b.

During the ESD process, the evaporation of the solvent causes the size of each droplet to decrease, while the amount of charge remains unchanged. Therefore, the surface charge density of the droplet increases. As the solvent further evaporates, there is stronger Coulomb repulsion between surface charges.

For an ideal droplet, the outward charge repulsion is:

(1) $$\varepsilon_{0}E^{2}/2,$$

where $\varepsilon_{0}$ is dielectric constant, *E* is electric field strength.

And the inward force cause by surface tension is:

(2) 2γ/R,

*γ* is the surface tension, *R* is the droplet radius.

When the Coulomb repulsive force of the surface charge is greater than or equal to the surface tension of the droplet, the Rayleigh limit is reached [25,26]

(3) $$\varepsilon_{0}E^{2}=2\gamma /R,$$

(4) E=(4γ∕ε0R)12

The droplet will lose its stability and break further, and the surface will emit smaller droplets with a smaller size but a high charge, which is called Coulomb fission [27].

Therefore, the droplets formed by the ESD process are usually smaller than than that available from conventional mechanical atomizers. And due to the unipolar charge characteristics of the fine droplets after atomization, Coulomb repulsion will occur between the droplets with the same kind of charge, so that agglomeration will not occur, the size distribution range of the droplets is much narrower.

In this paper, we use 10 mg/mL Ti_3_C_2_T_x_ /IPA dispersion as the solution for ESD. Thanks to the low boiling point and low viscosity of IPA, the solvent evaporates and droplets fission quickly during the electrostatic spray deposition process. The size of the droplets is rapidly reduced, and the surface charge density increases sharply, so that it can be fully atomized into Ti_3_C_2_T_x_ aerosol. Next, non-agglomerated nanoparticles are formed and the nanoparticles are adsorbed on the TPU fibers to form a solid sensing layer under the action of the Coulomb force.

On the other hand, we use a hollow template to cover the TPU electrospun membrane as the substrate, which changes the electric field distribution on the surface of the TPU membrane, thereby guiding the Ti_3_C_2_T_x_ aerosol formed by atomization to be selectively deposited under the action of the electric field to realize the patterned deposition of the sensing layer.

It is especially important that the typical experimental device of the ESD process is the same as the electrospinning process, which is fully compatible with the electrospinning manufacturing process of the sensor substrate in this work and the subsequent secondary electrospinning packaging process, and can be completed on the same device, which greatly simplifies the process procedure.

### 3.2. Electromechanical Behavior and Strain Sensing Mechanism Analysis 

**Figure 4:**
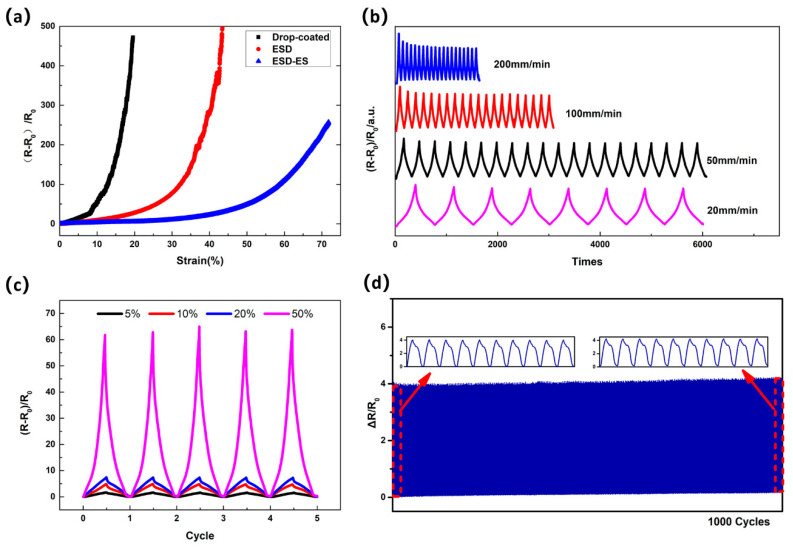
(**a**) The basic characteristics of the strain sensors. The relative resistance change versus the applied strain of up to 100%. Drop coated represents the characteristics of sensor with drop coated sensing layer; ESD represents the characteristics of sensor with ESD sensing layer but without sandwich structure of the electrospun encapsulation; ESD-ES represents the characteristics of sensor with ESD sensing layer with sandwich structure of the electrospun encapsulation. (**b**) the relative resistance variation of ESD-ES sensor with a strain of 30% at different strain rates; (**c**) the relative resistance change of ESD-ES sensor with various cyclic strains at a strain rate of 10 mm min^−1^; (**d**) the performance of the ESD-ES sensor under cyclic tensile loading for 1000 cycles.

Sensors produced by different deposition and encapsulation technique have been designed and prepared, respectively. We mainly prepared and compared three kinds of sensors: sensor with drop coated sensing layer; sensor with ESD sensing layer but without sandwich structure of the electrospun encapsulation; sensor with ESD sensing layer with sandwich structure of the electrospun encapsulation. The sensing performance of strain sensors were investigated (Figure 4a).

From the results of the test, the sensor with the drop-coated sensing layer has high sensitivity but the detection range is small (less than 20%). This could be understood by the disconnection of mechanical microcrack junctions under tensile stress in the Ti_3_C_2_T_x_ layers, followed by the rapid crack propagation of the stacked Ti_3_C_2_T_x_ layers under higher strains. When the strain reaches 20%, the resistance increases instantaneously, which can be attributed to the rupture of the sensing layer. By contrast, the sensor with ESD Ti_3_C_2_T_x_ sensing layer has high sensitivity, and the workable strain is significantly improved to 42% and has a good ability to detect a large range of strain. On the other hand, we use electrospinning technic to spin the TPU nanofiber on the surface of the sensing layer again, thereby sandwiching the Ti_3_C_2_T_x_ layer between the TPU nanofiber membranes. As can be seen from the Figure 4a, the ESD-ES sensor with sandwich like structure has a broader workable strain range (more than 70%). It can be contributed to the sandwich like structure of the electrospun encapsulation to more uniform stress distribution.

Figure 4b shows the relative resistance change of the ESD-ES strain sensor with a strain of 30% at different strain rates (20–200 mm min^−1^). Note that the electrical responses of the strain sensors exhibit stable durability at different strain rates, which may be attributed to the strong adhesion between ESD Ti_3_C_2_T_x_ MXene and ES TPU nanofiber membranes. Figure 4c shows the sensor response under different cyclic strains of 5%, 10%, 20%, and 50% with the same strain rate. The peak variations of the electrical signals were almost the same as the strain increase. The larger workable strain indicates that the ESD-ES strain sensor has potential applications for wearable electronic devices. For example, since the average elongation of human skin is 3% to 55% [28]. To evaluate the durability of this sensor, Figure 4d shows the performance of the sensor under a cyclic loading of 10% strain at a rate of 200 mm min^−1^ for 1000 cycles. The sensor after cyclical stretching still maintained an electrical performance (high stability and high sensitivity) similar to that before the cyclic stretching tests. The sensor exhibited stability during the cyclical loading and unloading processes. This repeatability test demonstrates that such a ESD-ES strain sensor has good durability.

In order to study the sensing mechanism, optical microscope (Nikon MM-400, Japan) and scanning electron microscope (SEM, TM3030, Hitachi, Japan) were used to observe the morphology of the MXene conductive sensing layer. The morphology of the Mxene layer prepared by the drop-coated method and ESD process were observed respectively before and after stretching.

**Figure 5:**
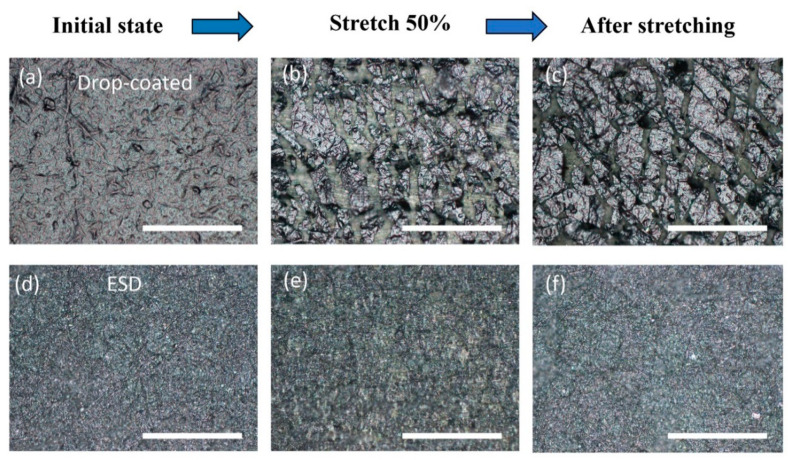
Optical images of drop coated sensing layer in the initial state (**a**) and stretched states by 50% (**b**) and after stretching (**c**); Optical images of ESD sensing layer in the initial state (**d**) and stretched states by 50% (**e**) and after stretching (**f**). The scale bar is 200 μm.

**Figure 6:**
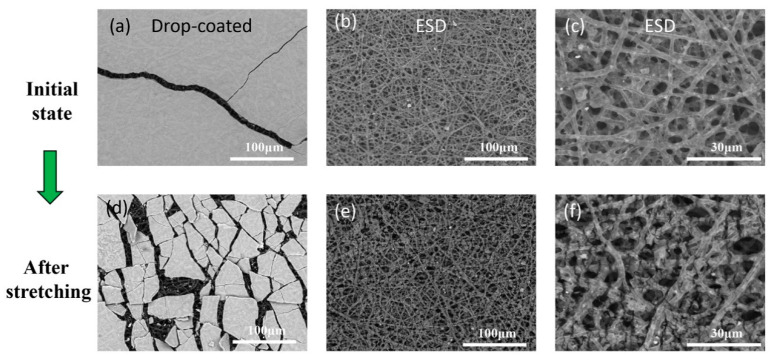
SEM images of drop coated sensing layer in the initial state (**a**) and after stretching (**d**); SEM images of ESD sensing layer in the initial state (**b,c**) and after stretching (**e,f**).

Figure 5a and Figure 6a shows that the MXene sensing layer prepared by the drop-coated method forms a block on the surface of the nanofiber membrane, and the surface is smooth. After stretching, brittle fracture occurs, forming a large number of cracks and discrete block, and obvious separation and shedding of nanofibers from the substrate, showing poor adhesion (Figure 5b,c and Figure 6d).

In contrast, a coating with good morphology is formed on the surface of the TPU fiber membrane through the ESD process, and the coating has strong bonding force and is not easy to break or fall off. The surface of the sensing layer is relatively dense, and the conductive material is better attached to the nanofibers in the hole (Figure 5d and Figure 6b,c). After stretching, microcracks can be seen on the fiber, but most of them can still maintain a good connection state (Figure 6e,f). This is consistent with the results of the stress-strain tensile test. Therefore, using the ESD process to prepare the sensing coating layer can significantly increase the tensile detection range of the sensor and improve the adhesion of the coating.

The MXene dispersion is ejected from the nozzle under the action of a high-voltage electric field. Due to its unipolar charge characteristics, Coulomb repulsion will occur between micro-droplets with the same charge, so that agglomeration does not occur. After atomization and drying, discrete non-agglomerated conductive micro-nano particles are formed. Then, because the nanofiber membrane as the receiving substrate is grounded, the electric potential is low. The conductive micro-nano particles are adsorbed and deposited on the nanofiber membrane under the action of Coulomb force. Thanks to the high porosity of the electrospun membrane and the adsorption of the Coulomb force, the MXene micro/nano conductive particles are adsorbed into the pores of the nanofiber network, forming a conductive layer on the fiber that is not easy to fall off. The ESD discrete state of MXene forms a conductive layer (Figure 6e) on the electrospun nanofiber grid, which is equivalent to forming a sensor grid composed of a large number of crisscrossed nanofiber strain sensors in different directions. When it is stretched, the stress distribution is more uniform.

**Figure 7:**
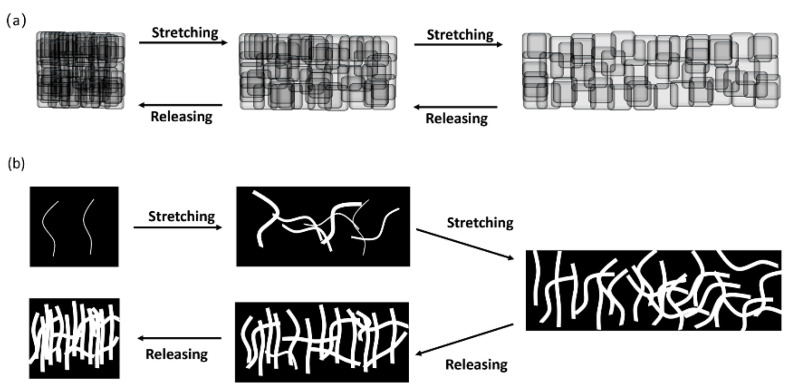
Schematic demonstration of the sensing mechanism of ESD sensing layer (**a**) and drop-coated layer (**b**).

Moreover, during the process of ESD, the charged aerosol carries the same electric charges and repels each other, the conductive particles produced after atomization and drying are adsorbed to the surface of the fiber in a discrete state. When stretched, the discrete MXene particles slippage can be generated to adapt to the partially applied stress and increase the strain, thereby continuing to maintain a better connection state, with better redundancy and adaptability, and avoiding the sharp rise in resistance caused by the occurrence of cracks. The proposed sensing mechanism is schematically demonstrated in Figure 7.

### 3.3. Practical Application in Motion Monitoring

In order to demonstrate the potential applications of the ESD strain sensors as wearable devices, we next evaluated the ability of the sensor to detect human activities. Sensors were attached to the neck, by virtue of the flexibility and widely workable strain range, a large range of motions of the human body, such as the motions of joints and relaxation of muscle, can be recognized by this sensor.

**Figure 8:**
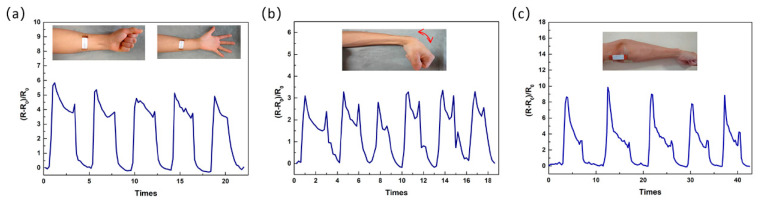
The monitoring of human motion using the ESD-ES strain sensors. The response to the motions of making a fist (**a**), wrist bending (**b**), and elbow bending (**c**).

As shown in Figure 8a, when the man begins to make a fist, the relative resistance change. When the fist is released, the relative resistance returns to the original value. Similar measurements are made on a cyclic bending wrist, and the strain sensor also shows good performance (Figure 8b). The sensory response to the cyclic motion of the cyclic bending wrist also indicates that the ESD-ES strain sensor is stable during the monitoring process. As shown in Figure 8c, the elbow motion can also be well recognized by the sensor. All the above results demonstrate that the ESD strain sensor has the ability to monitor the large deformation of the human body.

## 4. Conclusions

In this study, strain sensors with high sensitivity and broad sensing range was fabricated through the ESD-ES composite process. Thanks to the Coulomb repulsion of conductive particles with the same charge during the ESD process, agglomeration is avoided. The discrete MXene sheets slip to adapt partial strain during stretching. The crisscrossed porous structure of the nanofiber membrane helps to distribute the stress uniformly. Benefiting from these unique advantages, the workable strain range has been greatly improved (from 19% to 42%). Additional ES encapsulation sandwich structure help to improve the adhesion between Mxene layer and nanofiber membrane and more uniform stress distribution. Thus further increase the strain sensing range to 70%. The repeatability test (1000 cycles) demonstrates that ESD-ES strain sensor has good durability. Human motion detections demonstrate that ESD-ES sensors have wide application prospects in smart wearable device. This study provides a practical and meaningful strategy to fabricate highly stretchable and sensitive strain sensors for human motion monitoring.
